# Inhibition of serotonergic signaling induces higher consumption of both sucrose solution and toxic baits in carpenter ants

**DOI:** 10.1038/s41598-021-98030-8

**Published:** 2021-09-28

**Authors:** Roxana Josens, Alina Giacometti, Martin Giurfa

**Affiliations:** 1 Laboratorio de Insectos Sociales, Departamento de Biodiversidad y Biología Experimental and Instituto de Fisiología y Biología Molecular y Neurociencias (CONICET), Facultad de Ciencias Exactas y Naturales, Universidad de Buenos Aires. Ciudad Universitaria, Buenos Aires, C1428EGA Argentina; 2 Research Centre on Animal Cognition, Center for Integrative Biology, CNRS, University of Toulouse, 118 route de Narbonne, 31062 Toulouse Cedex 09, France; 3 Institut Universitaire de France (IUF), Paris, France

**Keywords:** Animal behaviour, Neuroscience, Feeding behaviour, Motivation, Reward, Environmental sciences

## Abstract

Biogenic amines play an important role in the regulation of appetitive responses in insects. Among them, serotonin (5-HT) regulates feeding-related processes in numerous insect species. In carpenter ants, 5-HT administration has been shown to depress feeding behavior, thus opening the possibility of using 5-HT modulation in control strategies against those species considered as pest. Here we studied if administration of a 5-HT antagonist, ketanserin, promotes feeding of a sucrose solution and a toxic bait in carpenter ants *Camponotus mus*. We found that 3 h after a single oral administration of ketanserin, the mass of sucrose solution consumed by carpenter ants increased significantly. A similar effect was found after a chronic administration that lasted 5 days. Yet, ketanserin did neither affect the intake rates nor the activity of the pharyngeal pump that mediates feeding dynamics. In addition, ketanserin promoted the consumption of a toxic bait based on boric acid. Our results thus show that feeding motivation and consumption of both sucrose solution and a toxic bait can be enhanced via prior administration of ketanserin. We discuss the possible mechanisms underlying these effects and conclude that understanding basic physiological and neural principles that underlie feeding motivation allows establishing more efficient control strategies for pest insects.

## Introduction

Pest insects constitute a global threat because of their significant negative effects on human socio-economic activities. Control strategies are necessary to eradicate them and/or to reduce the impact of the damages they produce. Such strategies need to be environmentally friendly and reduce the release of pesticides and other chemicals to the environment. This can be achieved by favoring the use of physical barriers, traps, and baits as control methods that are less harmful for human and environmental health.

Several ant species are regarded as pests because they interfere with human activities in urban, agricultural, and natural environments. A solution developed to control some of these species is the use of toxic baits that can have an impact at the colony level when ants transport them to the nest. Sugary baits have been used to target nectivorous ants since a large part of their diet involves sugary solutions obtained mainly from extrafloral nectaries and from honeydew excreted by hemipterans^1,2^. However, nectivorous ants reject commercial baits in many situations, which limits their effectiveness^3–5^. Thus, the use of these baits requires a deep knowledge of ant biology and behavior in order to achieve higher acceptance and food ingestion. To promote consumption of toxic baits, it is particularly relevant to focus on the physiological and neural processes that regulate food ingestion.

Biogenic amines play an important role in the regulation of appetitive responses in insects^6,7^. They act as neuromodulators of feeding-related behavior and may promote food ingestion. In honey bees, for instance, octopamine and tyramine mediate appetitive responsiveness to sucrose solutions and affect associative learning^8^. Serotonin (5-HT) deserves special attention in a feeding context as it has been shown to regulate feeding and feeding-related processes such as hunger, gut motility, and dieresis in numerous insect species, including crickets^9^, migratory locusts^10^, fall armyworms^11^, cabbage worms^12^, blowflies^13^, kissing bugs^14,15^, stick insects^16^, and honey bees^17^. In the carpenter ant *Camponotus mus*, the focus of our study, an increase in 5-HT levels via oral administration reduces feeding activity^18^ without modifying the acceptance threshold for sucrose. This decrease in feeding activity also involves a reduction of ingestion rate, mainly via the modulation of the pumping activity while feeding on nectar^18^.

With this in mind, we reasoned that the use of a 5-HT antagonist could have the opposite effect: i.e. promote feeding and increase food consumption. If this were the case, blocking the serotonergic system could be a valuable strategy for enhancing the consumption of toxic baits. Thus, we aimed at determining if prior administration of ketanserin, a powerful 5-HT inhibitor, increases subsequent consumption of sucrose solution and toxic baits in carpenter ants.

## Results

### Experiment 1: effect of an acute administration of ketanserin on sucrose feeding

In a first, experiment we tested the effect of an acute oral administration of ketanserin on a subsequent consumption of a sucrose solution offered in a foraging arena to which the treated ants had access between 3 and 6.5 h after treatment. This period was determined based on both a preliminary assay aimed at testing the efficacy of the ketanserin treatment and previous investigations on the action time of related amines^18^.

Two groups of 30 forager ants *C. mus* of similar weight were enclosed each in a container where they received a 22 µl drop of sucrose solution 30% (w/w), which represents a theoretical average volume of 0.75 µl per ant. During an initial *administration phase*, one group (control) experienced the pure sucrose solution while the other group (ketanserin) experienced the same sucrose solution spiked with ketanserin (2 mM). Considering this ketanserin concentration and the theoretical volume ingested per ant (0.75 µl; see above), the dose of ketanserin administered was 0.82 µg per ant. Ants consumed the drop entirely and shared their crop contents via trophallactic contacts. Thereafter (between 3 and 6.5 h post treatment; see above), ants of both groups were subjected to an *evaluation phase* during which each individual was placed in a feeding arena and was offered a small drop of sucrose solution, whose concentration (15% w/w) was lower than that used to deliver the ketanserin. In this way, we aimed at detecting fine modulations of feeding behaviors induced by this antagonist, which may occur at low-concentration ranges given the saturating effect induced by higher sucrose concentrations.

We weighed the ant before and after the feeding that occurred during the evaluation phase. Each ant was measured only once. We calculated the mass ingested (mg) as the difference between the final and the initial weight, and the feeding time (s). With both variables, it was possible to calculate the ingestion rate (mg/min) per ant. A rejection was counted whenever the mouthparts of an ant touched the drop and the difference between its initial and its final weight was less than 1% of the original weight. In this experiment only one ant of the control group rejected the sucrose drop offered during the evaluation phase (control: 1 from 32 ants; ketanserin: 0 from 42 ants), thus showing that no differences existed between groups in terms of food rejection (Fisher exact test; *P* = 0.43). This result indicates, therefore, that the ingestion of ketanserin did not affect the predisposition of ants to engage in subsequent feeding.

**Figure 1:**
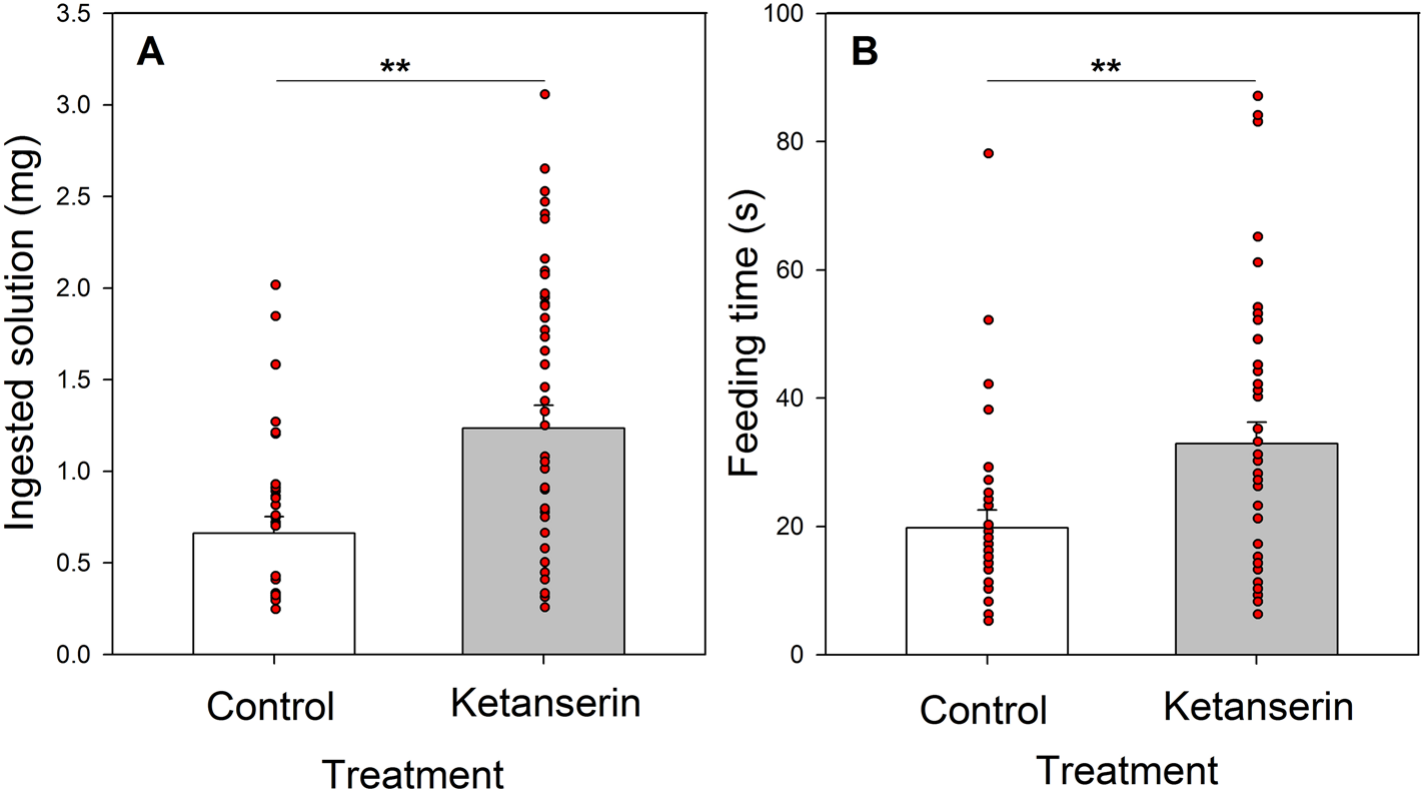
Feeding behavior during the effect period. (**A**) Ingested solution (mg) and (**B**) Feeding time (s) for each treatment: Control (white) and Ketanserin (gray). Bars represent the means and the s.e. and red dots indicate individual ants. **: *P* < 0.01.

The analysis of the ants that fed during the evaluation phase showed that ants in both groups had a similar initial weight before the feeding event (*t-test*: t =  − 0.3; N = 73; *P* = 0.765). Yet, the amount of solution ingested by the ants treated with ketanserin was significantly higher than that ingested by the control ants (*t-test*: t = 3.13; N = 73, *P* = 0.0025. Fig. 1A). In addition, they spent more time feeding than the control ants (*t-test*: t = 2.83; N = 73, *P* = 0.006. Fig. 1B).

The ingestion rate (i.e. the mg of sucrose solution ingested per min) was similar for both groups of ants. For the control group it was 1.98 ± 0.14 mg/min (mean ± se; N = 32) and for the ketanserin group 2.22 ± 0.11 mg/min (mean ± se; N = 42). These values did not differ significantly from each other (*t-test*: t = 1.39; N = 73, *P* = 0.169). Thus, ketanserin increased the mass of sucrose solution ingested and prolonged the time spent feeding while leaving the ingestion rate unaffected.

### Experiment 2: effect of a chronic administration of ketanserin on sucrose feeding

Given the results observed after an acute administration of ketanserin, we aimed at determining if a chronic administration of ketanserin would affect in a longer delay (i.e. 18 h after the ketanserin administration) the consumption of sucrose solution.

To answer this question, we placed two groups of 10 ants of similar weight in containers (*t-Test* analysis for ant weight: *t* = 0.23; N = 44; *P* = 0.82) and fed them during five consecutive days, either with a 30% w/w sucrose solution (control group) or with a 30% w/w sucrose solution spiked with 2 mM ketanserin (ketanserin group). Ants were given an amount of solution equivalent to 0.4 µl per ant per day during five days. On the sixth day, and more specifically 18 h after the administration phase, ants of both groups were subjected to the evaluation phase. Each ant was then offered a drop of a sucrose solution (10% w/w). As explained before, the lower concentration of sucrose (with respect to that experienced during the administration phase) was chosen to detect fine feeding modulations by ketanserin, which could remain hidden at higher concentrations. Ants from both groups alternated in the feeding arena. As in the previous experiment, we determined the number of ants that rejected feeding in both groups. In the control group, 7 out from 28 ants rejected the solution in the evaluation phase, while in the ketanserine group, 5 out from 28 ants did so. These proportions did not vary significantly from each other (Fisher exact test; *P* = 0.75). This shows that the chronic administration of ketanserin did not change significantly the willingness of ants to feed on the sucrose solution.

**Figure 2:**
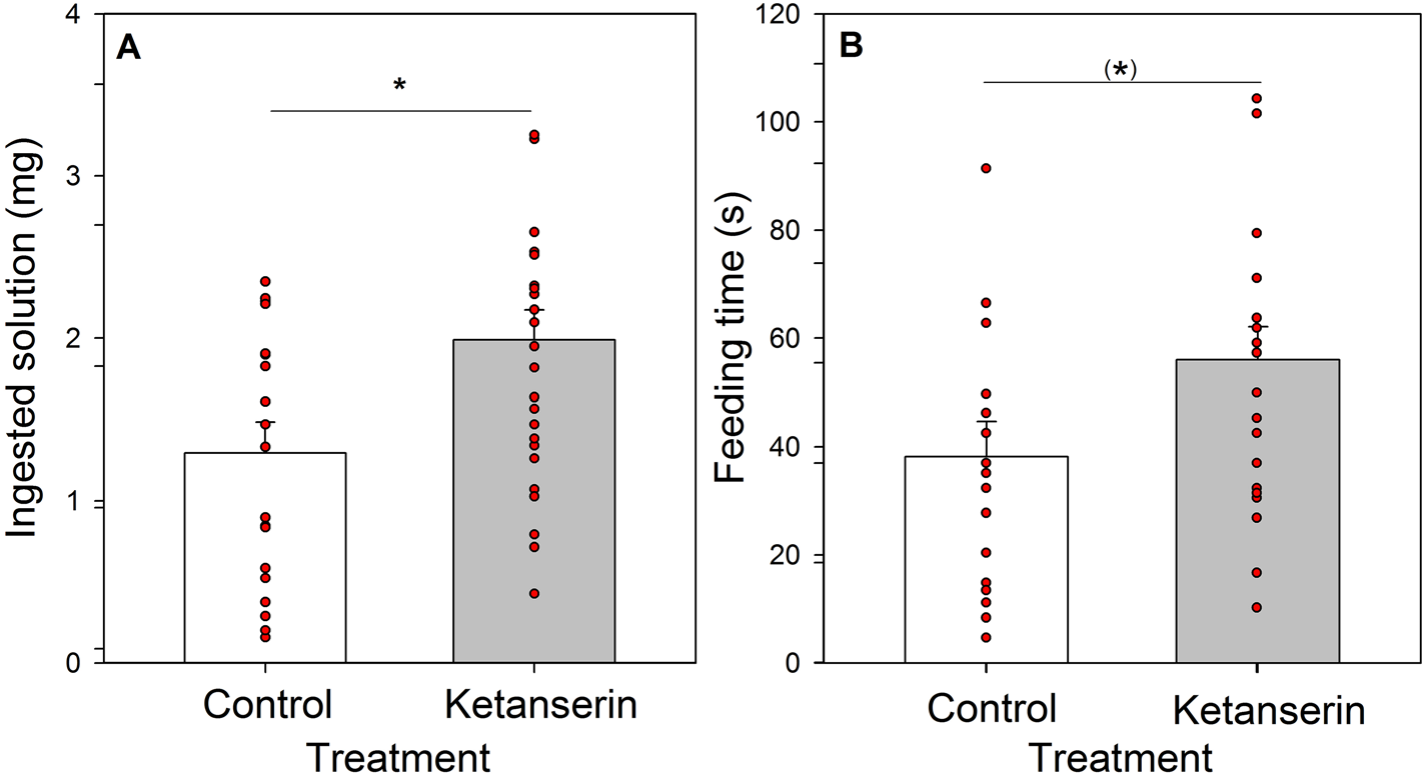
Feeding behavior of ants on the 6th day following a 5-day chronic administration of pure sucrose solution or sucrose solution with ketanserin. (**A**) Ingested solution (mg) and (**B**) Feeding time (s) for each treatment: Control (white) and Ketanserin (gray). Bars represent the means and the s.e. and red dots indicate individual ants. *: *P* < 0.05; (*): *P* = 0.053.

Feeding variables were analyzed on the ants that fed on the sucrose solution 18 h after the last drug administration. Ants that received ketanserin during the five consecutive days ingested more solution than the control group (*t-Test*: *t* = 2.62; N = 44; *P* = 0.012. Fig. 2A). Feeding time tended to be longer for the ketanserin group, yet the difference did not reach significance (*t-Test*: *t* = 2; N = 36; *P* = 0.0537. Fig. 2B). Thus, after a chronic administration of ketanserin, a long-delay, significant effect on the feeding behavior (amount of solution ingested) was observed.

The intake rate of the control group (2.25 ± 0.26 mg/min; mean ± s.e.; N = 16) and that of the ketanserin group (2.21 ± 0.17 mg/min; mean ± s.e.; N = 20) did not differ statistically (*t-Test*: *t* = 0.14; N = 36; *P* = 0.89). Considering that intake rates depend on the activity of the pharyngeal pump^19,20^, and that chronic administration of 5-HT affects more the pumping frequency than an acute administration^18^, in this particular experiment, we performed non-invasive recordings of the pumping frequency while the ants fed undisturbed in the arena. To this end, we adapted the feeding arena to allow recordings of the pumping frequency. A micro centrifuge tube containing ad libitum sucrose solution was inserted in the center of the arena, with a surrounding area covered with a wet filter paper with a metallic net laying on top of it. One electrode was in contact with the solution while the other electrode was fixed to the metallic net. In this way, when an ant stood on the metallic net and contacted the solution with her mouthparts, the circuit was closed, allowing the recording of the electrical activity generated by the ant’s muscles during feeding. For both ketanserin- and control ants, we quantified the instantaneous pumping frequencies during intake at three time points corresponding to 0, 10, and 20 s following the beginning of intake.

**Figure 3:**
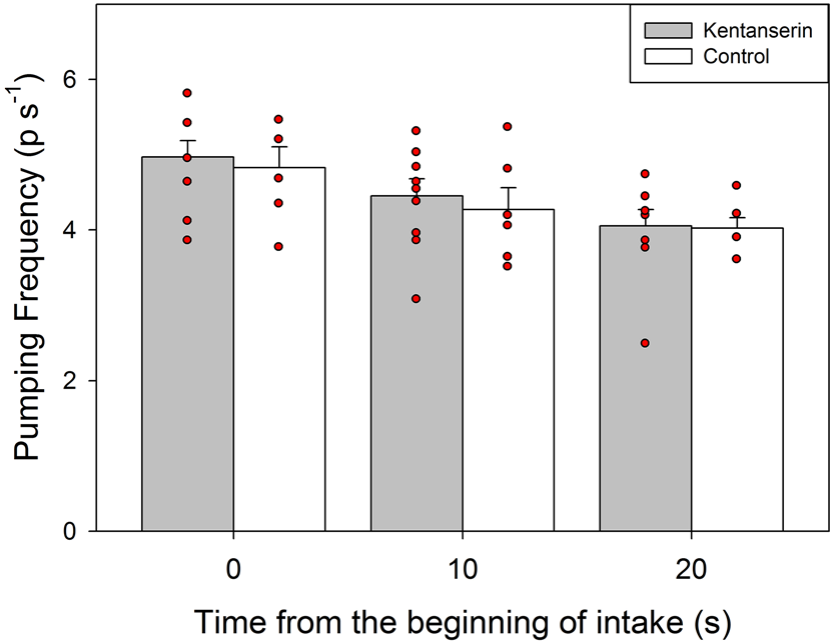
Instantaneous pumping frequencies (in peak/s; mean + s.e.) of Control (white) and Ketanserin (gray) treated ants feeding on sucrose solution at the beginning of the intake, and at around 10 s and 20 s from the beginning of the intake. Each point represents the average pumping frequency of an individual ant calculated as the average of eight consecutive frequency values measured every 0.64 s around 0 (0–5.12 s), 10 (7.68–12.16 s), and 20 s (17.92–22.4 s) from the beginning of intake. Different letters indicate significant differences (*p* < 0.05).

In agreement with the behavioral results obtained for the intake rate, no effect of ketanserin was observed on the activity of the pharyngeal pump. Pumping frequencies were similar between both groups of ants (*Treatment:* F = 0.16; *P* = 0.7; see Fig. 3) for the three recorded times (*Treatment* × *Time:* F = 0.13; *P* = 0.9). They only varied with time, decreasing throughout the intake process (*Time:* F = 18.13; *P* = 0.001).

### Experiment 3: effect of an acute administration of ketanserin on toxic-bait feeding

In a final experiment, we assessed the effect of ketanserin on the consumption of sucrose solution spiked with an aversive substance. To this end, we added boric acid (4% w/v) to a 20% (w/w) sucrose solution offered during the evaluation phase, as it deters *C. mus* ants from food sources. The choice of this compound was further justified by its use in commercial baits employed for ant control so that our experiment studied if 5-HT inhibition promotes aversive-bait consumption in *C. mus* ants.

In this case, the administration and the evaluation phases were similar to those described for Experiment 1 (see above) with the difference that the administration phase was done on an individual basis (i.e. a single ant enclosed in a container with a 0.75 µl drop of sucrose solution, containing or not 2 mM ketanserin). In addition, the evaluation phase occurred between 3 and 4.5 h post treatment, and the sucrose solution offered during the evaluation phase contained boric acid. The concentration of the sucrose solution was increased to 20% to promote feeding even in the presence of the deterrent boric acid. The concentration of boric (4%) was higher than values commonly reported for toxic baits used against insect pests (between 0.5 and 2%)^21^ to determine if ketanserin was able to overcome the rejection induced by such a high boric-acid content.

The number of ants that rejected the food spiked with boric acid during the evaluation phase did not differ between the control and the ketanserin group. In the control group, 8 out of 41 ants rejected the solution while in the ketanserin group, 10 out of 40 ants did so. These proportions did not differ statistically (*Fisher exact test*; *P* = 0.60). Thus, we confirmed again that the ingestion of ketanserin had no effect on the ants’ willingness to feed subsequently on the sucrose solution spiked with boric acid.

**Figure 4:**
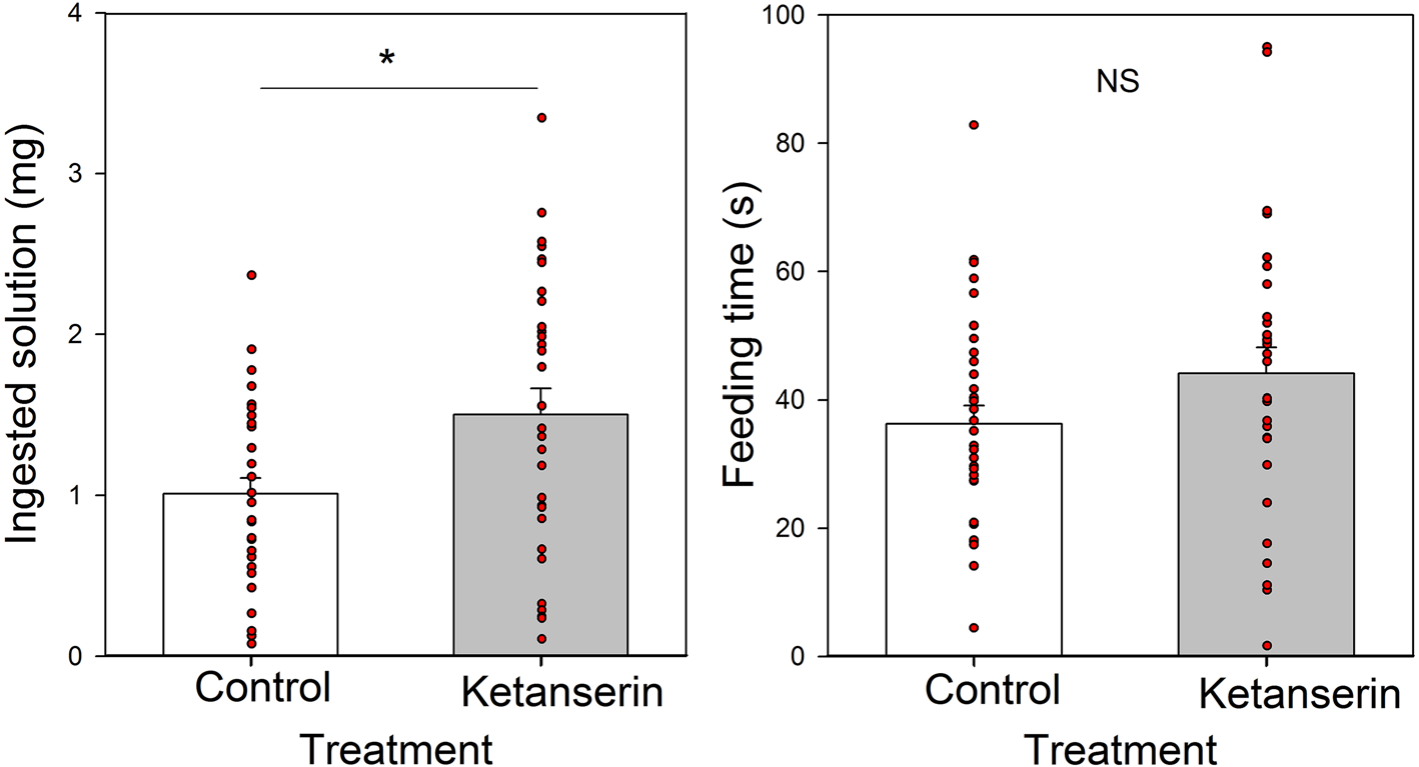
Feeding behavior of ants feeding on a toxic bait (sucrose solution 20% w/w added with boric acid 4% w/v) during the effect period. **A** Ingested solution (mg) and **B** Feeding time (s) for each treatment: Control (white) and Ketanserin (gray). Bars represent the means and the s.e. and red dots indicate individual ants. NS: not significant; *: *P* < 0.05.

The initial weight of ants engaging in feeding during the evaluation phase did not differ between the control and the ketanserin group (*t-Test*: t = 0.89, N = 63; *P* = 0.377). Again, ketanserin-treated ants ingested more toxic solution than control ants (*t-Test*: t = 2.23 , N = 63; *P* = 0.029. Fig. 4A), even if both groups spent a similar time feeding on both solutions (*t-Test*: t = 1.60, N = 63; *P* = 0.115. Fig. 4B). Intake rates (mg/min) were slightly higher in ketanserin ants (2.12 ± 0.16 mg/min; mean ± se; N = 30) than in control ants (1.72 ± 0.15 mg/min; mean ± se; N = 33) but the difference was not significant (*t-Test*: t = 1.82, N = 63; *P* = 0.0737).

**Figure 5:**
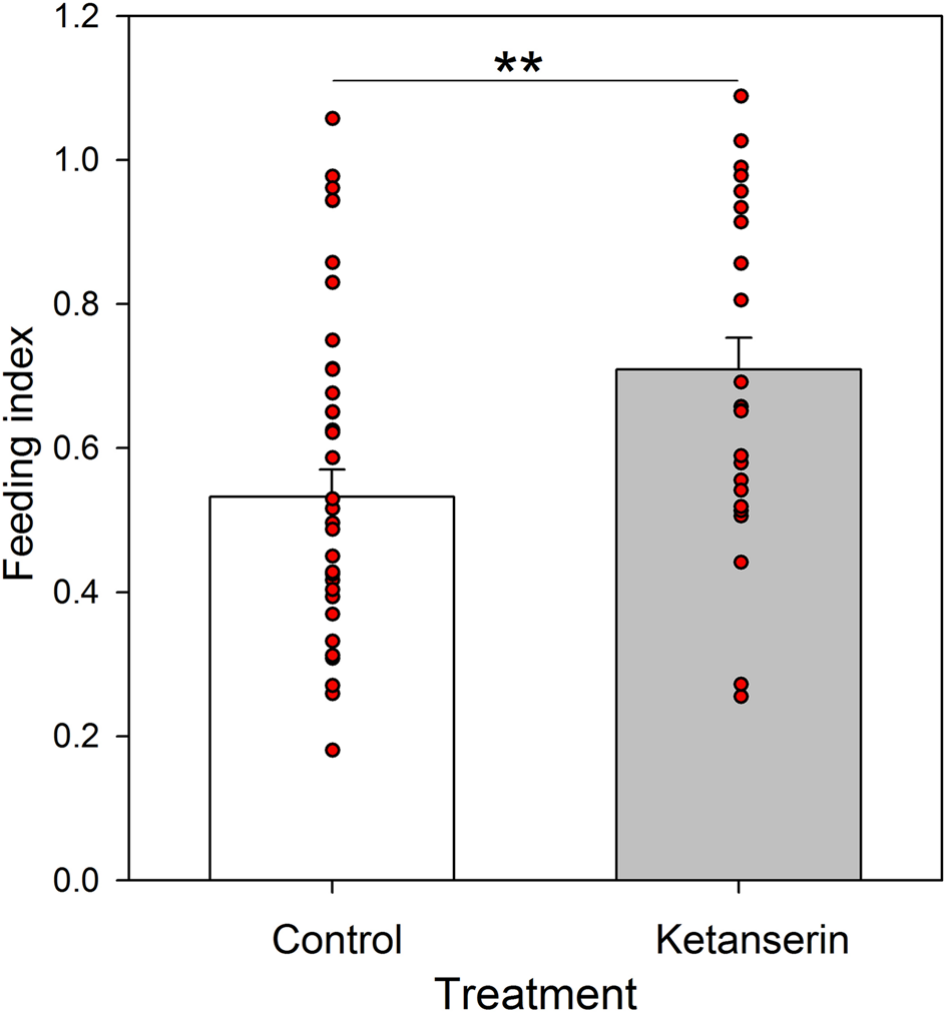
Feeding index [feeding time/(feeding time + interruption time)] for each treatment: Control (white) and Ketanserin (gray). Bars represent the means and the s.e. and red dots indicate individual ants. Red dashed line indicates the mean. **: *P* < 0.01.

Ants frequently interrupt their feeding when feeding on a low-concentration solution^22^, an aversive food, or a solution with low palatability (i.e. sucrose solution spiked with a bitter compound, Prina and Josens, in prep). As the toxic bait belongs to the latter category, we recorded feeding interruptions and calculated a feeding index [feeding time/(feeding time + interruption time)]. A feeding index equal to 1 corresponds to an ant that fed without interruptions. On the contrary, smaller index values reflect feeding with longer interruptions. The feeding index was higher in the ketanserin group than in the control group (*t-Test*: t = 3.03, N = 63; *P* = 0.0036. Fig. 5), thus revealing a higher acceptance of the solution with the toxic bait after ketanserin treatment.

## Discussion

Our study analyzed the relationship between feeding behavior and serotonergic signaling in the carpenter ant *Camponotus mus.* To this end, we studied whether a 5HT-receptor antagonist, ketanserin, affected the propensity to consume both sucrose solution and sucrose solution spiked with boric acid, an aversive substance used in pest control strategies^23–28^. Our results show that ketanserin did not affect the motivation of ants to feed upon contact with the sucrose solution offered during the evaluation phase. Yet, it changed the ingestion behavior itself as the consumption of sucrose solution offered during that phase increased 3 h after oral administration of ketanserin. In addition, chronic consumption of the antagonist induced enhanced feeding, which was sustained even 18 h after the last drug administration. Short-term consumption of ketanserin also promoted feeding, counteracting the aversion for the boric acid contained in the sucrose solution. Taken together, these results indicate that serotonergic signaling inhibits feeding behavior and that this effect can be suppressed by administering ketanserin.

Ketanserin in sucrose solution affected feeding behavior 3 h after its ingestion. Thus, the process by which ketanserin effectively inhibits 5-HT receptors after oral ingestion requires time. This period coincides with the period of 3.5 h found in a previous work in which 5-HT administered orally in sucrose solution had the opposite effects to those produced by ketanserin on feeding variables^18^. In honeybees, the impact of different biogenic amines on appetitive responses is generally evaluated within the first 30 or 60 min after oral treatment^29–31^. However, to our knowledge, experiments that address the effect of oral administration of 5-HT or 5-HT antagonists on appetitive responsiveness in bees are missing. In the ant *Odontomachus kuroiwae,* changes in aggression behavior are detectable 1 h after oral administration of ketanserin but the effects tend to increase during the following hours^32^.

Amongst the feeding variables considered in this study, the mass of solution ingested was the only one consistently modulated by ketanserin. In the different experiments performed, the ketanserin-treated group showed a significant increase in this variable when compared to the control group. As in each experiment ants of both groups had similar sizes, differences in the mass of solution ingested can only be attributed to the administration of ketanserin. This result is consistent with the feeding promoting effect of other 5-HT antagonists found both in vertebrates^33^ and in other insects^34–36^. For instance, honey bees injected with methiothepin recovered both appetitive responses and appetitive olfactory memories previously impaired via a conditioning-taste aversion protocol^17^.

The time spent feeding also increased after the ingestion of ketanserin, yet not always significantly. This result agrees with the fact that, in *C. mus* ants, feeding time is not always subject to modulation. Factors such as the productivity of the food source, the size of the ant, or the colony's state and nutritional requirements^19,37^ have more consistent effects on the mass of food ingested than on feeding time. Furthermore, the lack of effect of ketanserin on feeding time agrees with the fact that orally administered 5-HT does not affect this variable either^18^.

Ants treated with ketanserin showed a similar intake rate to control ants. Consistently, the rhythmical contractions of the sucking pump (pharyngeal pump) recorded in Experiment 2 were similar between both groups. Differences were neither observed at the beginning of the intake nor after 10 or 20 s. This was unexpected because oral administration of 5-HT decreased the intake rate of sucrose solution, mainly via a reduction of the volume of solution per pump contraction, without modifying the sucrose acceptance threshold^18^. The pumping frequency diminished significantly only after a chronic treatment with 5-HT in sucrose solution during 6 consecutive days^18^. Based on these results, it could have been predicted that ketanserin would have the opposite effect on intake rate, i.e., increasing the intake rate of sucrose solution by increasing the volume per pump contraction or the pumping frequency after a 5-day chronic treatment (Experiment 2). Yet no effect of ketanserin was detected. One explanation for this might be related to the specificity of ketanserin as a 5-HT antagonist, which might not efficiently target the 5-HT receptors involved in the control of the activity of the sucking-pump. Pumping muscles controlling the intake rate do not express 5-HT receptors^18^, thus supporting the hypothesis that the decrease of intake rate after oral administration of 5-HT may occur via alternative pathways.

Serotonin regulates food intake in many insect species, e.g., flies^34,36^, bees^38^, ants^18^, cockroaches^35^, moths^39^, blood-sucking bugs^14,40^, mosquitoes^41,42^, aphids^43^, locusts^44^. Serotonin acts by binding to specific receptors that in mammals belong to seven families (5-HT_1_ to 5-HT_7_) characterized according to their sequence similarities, gene organization, pharmacological properties, signaling pathways, and reactions they trigger^45,46^. Orthologs of three receptor families (S1, S2, and S7) have been found in insects^47^.

Ketanserin is selective for 5-HT_2_ receptors^47,48^. The fact that the modulation of responses by ketanserin was generally opposite to that observed after 5-HT oral administration^18^ indicates a fundamental role of 5-HT receptors of the S2 family in key centers participating in the regulation of feeding activity. In *Camponotus* ants, information on 5-HT receptors is available for *C. floridanus*^49^, for which five different 5-HT receptor genes have been reported: *GPR5ht*_*7*_*, GPR5ht*_*1b*_*, GPR5htorph*_*2*_ (with a splicing variant), and *GPR5ht*_*2a*_. It is tempting to propose a specific role of the two latter receptor types in the feeding processes studied in our work given the recurrent involvement of 5-HT_2_ receptors in feeding regulation and their targeting by ketanserin. Further pharmacological studies are necessary to determine the receptor-binding specificity of ketanserin in *C. mus* ants.

In the last decades, strategies to control pests, including some ant species, have attempted to reduce the amount of pesticides used to minimize the impact on the environment. Baiting is an effective and environmentally friendly alternative for managing pest ants^50^ as it avoids affecting non-target insects for which the designed baits are unattractive or less accessible. To this end, studies providing a deeper understanding of behavioral, physiological, and neurobiological mechanisms of decision-making processes in ants are necessary. As baits are sometimes rejected or under-consumed, information that helps to increase the acceptance of a toxic bait is important. For instance, the decision making of ants can be manipulated with a simple pre-baiting step^51^. Pre-baiting can increase the bait acceptance of ants via social information transfer about the location, safety, or value of the resource^52^. Other studies have shown that synthetic ant pheromones can be used to promote bait consumption by Argentine ants, *Linepithema humile*, either through an increase in the number of feeding ants^53^ or via an enhancement of the subjective value of the reward encountered^54^. These results indicate that the combination of pheromones and toxic baits administered in association with food constitutes a selective and efficient strategy to control pest ants^53,55^.

In the same way, the increase of toxic bait consumption following a pre-baiting acute consumption of sucrose solution containing a 5-HT antagonist could be useful to elaborate efficient control strategies against pest insects. Although using ketanserin in a massive scale could be difficult due to economic costs, the main message of our work is that strategies that aim to modulate neurotransmitter levels used in pre-baiting may be valuable and innovative ways to achieve greater bait acceptance and more efficient pest control. Studies combining neurobiological and behavioral analyses of feeding behavior in ants will pave the way towards this end.

## Materials and methods

### Insects

Three colonies of *Camponotus mus* (Roger) composed of more than 1000 workers and at least one queen were used in the experiments. The colonies were captured in Francisco Alvarez, Prov. Buenos Aires (Argentina). They were reared in the laboratory in artificial nests. Each nest consisted of a plastic box (30 cm × 50 cm × 30 cm) with a base coated with plaster and walls painted with fluon to prevent animals from escaping. Colonies were housed in piled acrylic plates and workers had access to freshwater, honey-water, and chopped insects within the container. Nests were maintained in the laboratory for 1 year under natural light/dark cycles and nearly constant temperature (23 ± 3 °C). Before the experiments, colonies were submitted to a carbohydrate reduction in the colony diet to increase feeding motivation^56^.

### Experiments

#### Experiment 1: effect of an acute administration of ketanserin on sucrose feeding

*Administration phase*: Two groups of 30 *C. mus* foragers of similar weight were each enclosed in a container (13.5 cm in diameter and 8 cm high) for ca. 30 min. A 22 µl drop of 30% sucrose solution was offered in the container. One group (control) experienced the pure sucrose solution while the other group (ketanserin) experienced the same sucrose solution spiked with ketanserin, an inhibitor of 5-HT receptors. Given the number of ants and the volume of sucrose solution offered, the theoretical consumption per ant was 0.75 µl.

Here and in the subsequent experiments, ketanserin was always delivered in a 30% sucrose solution because of the necessity of increasing its acceptance rate in order to evaluate its effect a posteriori. We thus chose a concentration of sucrose solution that is preferred by *C. mus* ants^22^. This strategy proved already to be successful in a work in which 5-HT was delivered in 30% sucrose solution to ants^18^ of the same species. Ketanserin was obtained from Sigma Aldrich [ketanserin (+)-tartrate salt > 97%. Product number S006]. The concentration of ketanserin present in the sucrose solution was 2 mM so that the dose of ketanserin ingested per ant in a volume of 0.75 µl was 0.82 µg. The treatment was considered to be finished when the food delivered in the container had been entirely consumed. Food delivery by trophallactic contacts was observed in all groups.

*Evaluation phase:* Between 3 and 6.5 h after the administration phase, each focal ant was gently enclosed in a vial and weighed on an electronic balance (Mettler-Toledo, resolution of 0.01 mg). It was then immediately placed on a wooden bridge (10 cm × 1.5 cm) leading to a feeding arena (2 cm × 1.5 cm) that offered in its center a drop of 15% (w/w) sucrose solution. The period chosen is based on both a preliminarily assay aimed at characterizing the period during which the ketanserin treatment would affect feeding and reported periods for the action time of biogenic amines^18^. A lower sucrose concentration was used in the evaluation phase with respect to the administration phase in order to detect fine modulations of behavioral variables. Previous work has shown that better modulation can be observed in ants for concentrations ranges similar to the one chosen here^54^ because higher concentrations induce saturation of some behavioral variables^57^.

Once the ant discovered the drop by itself, it was allowed to feed on it undisturbed. When the ant departed towards the bridge, it was captured and weighed again in the same vial. In this way, its weight after feeding could be compared to its initial weight. Afterwards, it was sacrificed. Ants from the control and the ketanserin group alternated in their access to the feeding arena during this procedure. To determine if the ketanserin treatment affected the willingness of the ants to engage in feeding during the evaluation phase, we recorded the number of ants that rejected the food offered during the evaluation phase. Rejection occurred whenever the ant touched the drop but the difference between its initial and its final weight was less than 1% of the original weight.

At the beginning of every recording day and before the start of the evaluation phase, a few workers were allowed to forage in the arena, stimulated by the presence of a drop of sucrose solution. In this way, the wooden bridge was already marked with trail pheromone for the evaluation phase from the first focal ant of the day, so all focal ants found the bridge with trail pheromones.

In this first experiment, we aimed at determining whether the ketanserin treatment affected feeding behavior with respect of that exhibited by a control group. For each ant that accepted the sucrose solution, we determined: (1) the mass of solution ingested (mg), which was obtained from the difference in weight after and before feeding on the sucrose drop; (2) the feeding time (s), which was measured as the time the ant spent with its mouthparts in contact with the drop of sucrose solution; and (3) the intake rate (mg/min), which is the quotient between the mass ingested and the feeding time.

#### Experiment 2: effect of a chronic administration of ketanserin on sucrose feeding

To study whether a prolonged exposure to ketanserin increases feeding motivation, two groups of 10 foragers of similar weight were captured from a nest and separated in two containers (13.5 cm in diameter and 8 cm in high) where they were provided with water ad libitum. During five consecutive days, one group (control) was fed with a 30% sucrose solution (w/w) while the other group (ketanserin) was fed with a 30% sucrose solution (w/w) containing 2 mM ketanserin. Given the volume offered to each group and the number of ants per group, each ant received a theoretical average of 0.4 µl per day. Food access ended at 18:00 of the fifth day.

At 12:00 of the sixth day, i.e., 18 h after the end of the administration phase, ants were weighted to obtain their initial weight and then allowed to enter into the feeding arena described previously for the evaluation phase. They were then offered a drop of a 10% sucrose solution (w/w) and for those ants that accepted the drop of sucrose solution, the same variables as in the previous experiments were quantified: the mass of ingested solution (mg), the feeding time (s), and the intake rate (mg/min). Control and ketanserin ants alternated in their visit to the arena.

The last ants in this experiment (i.e. in the last days of data recording) were tested in a particular arena that allowed recording the activity of the sucking pump while ants were feeding^19^. These ants were individually placed on a wooden bridge that led to the recording arena, which consisted of a surface of 2 cm × 2 cm covered with a wet filter paper and a metallic net covered with a thin layer of conductor gel on top of it. A micro centrifuge tube containing ad libitum sucrose solution was inserted in the center of the arena in such a way that the solution was never in contact with the metallic net. One electrode was in contact with the solution through a small lateral hole in the tube, while the other electrode was fixed to the metallic net. In this way, when an ant stood on the metallic net and contacted the solution with her mouthparts, the circuit was closed, allowing the recording of the electrical activity generated by the ant’s muscles during feeding. The signals were amplified 210 times and filtered using a band-pass filter (0.4–17 Hz, 3 dB). Recordings were observed and stored in a PC using an analogical-digital plate (ADC-212, Pico Technology Limited, St. Neots, Cambridgeshire, UK). All recordings were obtained using a 200 Hz sampling rate. Frequency values were then calculated every 0.64 s as defined by the recording program.

To compare the instantaneous frequencies during intake between treatments, we chose three time points occurring at 0, 10, and 20 s after the beginning of intake. We did not choose times beyond 20 s because some feeding events finished or tended to finish at that time, and the variability of signals increased at the end of the intake process. To characterize the pumping frequency for each time point, we averaged 8 frequency values measured consecutively every 0.64 s, covering a 5.12 s period in each case: from 0 to 5.12 s for the 0 s time point, from 7.68 to 12.16 s for the 10 s time point, and from 17.92 to 22.4 s for the 20 s time point.

#### Experiment 3: effect of an acute administration of ketanserin on toxic-bait feeding

We studied if the promoting effect of ketanserin on feeding would induce the ingestion of less palatable food. We focused in particular on responses to sucrose solution containing boric acid, a compound with proven deterrent properties for these ants^58^.

Forager ants of similar weight were captured from a nest and separated in individual containers (4.5 cm in diameter and 3.5 cm high). After thirty minutes of acclimatization, half of the ants were offered 0.75 µl of a 30% sucrose solution (w/w; Control) while the other half received 0.75 µl of a 30% w/w sucrose solution containing 2 mM ketanserin (Ketanserin). Only the ants that consumed entirely the offered solution were used in the experiment. Three hours after administration the recordings started and continued for 1.5 h more (4.5 h after administration). Ants were allowed to visit individually the feeding arena where they were offered a drop of a 20% sucrose solution (w/w) containing 4% boric acid (w/w). This choice corresponded approximately to the typical concentrations that can be found in sugary baits^59^ containing boric acid that are used for managing ants^23,28^ and other insect species^60^. We increased the concentration of boric (4%) with respect to common values to assess if ketanserin was able to overcome rejection induced by such a high boric-acid content. As in the previous experiments, we quantified the mass of solution (mg) ingested by each ant and the time (s) spent feeding (see above).

### Statistical analysis

The proportions of ants of both groups (control and ketanserin-treated) that rejected the food offered during the evaluation phase were compared using a Fisher Exact Probability Test. Subsequent analyses were performed on ants that actually engaged in feeding during that phase. Feeding variables (ingested solution, feeding time) were checked for normality and homoscedasticity by means of a Chi-square goodness test and a Levene test, respectively. Data were transformed for normality when necessary: the square root transformation was used for the mass of ingested solution in Experiments 1 and 3, and the log transformation was used for the feeding time in Experiment 1. Differences between ketanserin and control groups were evaluated using a two-sample t-test for all behavioral variables. In experiment 3, two outliers were eliminated (one for each treatment) as their feeding time was out of the range observable in all experiments. In this experiment, the differences in sample size between the mass of ingested solution and the feeding time are due to a data loss during the recording of feeding times. In consequence; the sample size corresponding to the intake rate was also smaller. The pumping frequencies of Experiment 2 were analyzed by means of a Repeated Measured ANOVA. The significance level used was 5% in all cases (two-tailed tests).
